# Evaluating the Reliability of Non-Specialist Observers in the Behavioural Assessment of Semi-Captive Asian Elephant Welfare

**DOI:** 10.3390/ani10010167

**Published:** 2020-01-18

**Authors:** Jonathan L. Webb, Jennie A. H. Crawley, Martin W. Seltmann, Océane Liehrmann, Nicola Hemmings, U Kyaw Nyein, Htoo Htoo Aung, Win Htut, Virpi Lummaa, Mirkka Lahdenperä

**Affiliations:** 1 Department of Animal and Plant Sciences, University of Sheffield, Sheffield S10 2TN, UK; n.hemmings@sheffield.ac.uk; 2 School of Psychology, University of Auckland, 23 Symonds Street, Auckland 1010, New Zealand; 3 Department of Biology, University of Turku, 20500 Turku, Finland; jahcra@utu.fi (J.A.H.C.); martin.seltmann@utu.fi (M.W.S.); oceane.liehrmann@gmail.com (O.L.); virpi.lummaa@utu.fi (V.L.); mirkka.lahdenpera@utu.fi (M.L.); 4 UFR des Sciences, University of Caen, 14000 Caen, France; 5 Myanma Timber Enterprise, Gyogone Forest Compound, Bayint Naung Road, Insein Township, Yangon 11011, Myanmar; kyaw.nyein.mte@gmail.com (U.K.N.); kohtooaung723@gmail.com (H.H.A.); winhtut641@gmail.com (W.H.)

**Keywords:** animal welfare, ethogram, behavioural assessment, stress, reliability, observational study

## Abstract

**Simple Summary:**

It is essential that elephant workers monitor the stress levels of their animals to uphold high standards of welfare. This can be done quickly and efficiently by observing elephant behaviour, however, the consistency of this approach is likely to vary between workers. While this variation has been tested in zoo elephants when observations were carried out by experienced observers, the consistency of observations made by non-experienced observers on the much larger population of Asian elephants working in Southeast Asia has yet to be explored. By constructing a list of elephant working behaviours, we employed three volunteer observers with no experience of elephant research to record the behaviour of Asian elephants working in Myanmar. We then tested the similarity between observations collected by the three observers, as well as the consistency that individual observers could repeatedly recognise the same behaviour. Overall, observers recognised the same behaviour from the videos and were highly consistent across repeated observations. These results suggest that the behaviours tested may represent useful indicators for welfare assessment, and that non-experienced observers can meaningfully contribute to the monitoring of elephant welfare.

**Abstract:**

Recognising stress is an important component in maintaining the welfare of captive animal populations, and behavioural observation provides a rapid and non-invasive method to do this. Despite substantial testing in zoo elephants, there has been relatively little interest in the application of behavioural assessments to the much larger working populations of Asian elephants across Southeast Asia, which are managed by workers possessing a broad range of behavioural knowledge. Here, we developed a new ethogram of potential stress- and work-related behaviour for a semi-captive population of Asian elephants. We then used this to collect observations from video footage of over 100 elephants and evaluated the reliability of behavioural welfare assessments carried out by non-specialist observers. From observations carried out by different raters with no prior experience of elephant research or management, we tested the reliability of observations between-observers, to assess the general inter-observer agreement, and within-observers, to assess the consistency in behaviour identification. The majority of ethogram behaviours were highly reliable both between- and within-observers, suggesting that overall, behaviour was highly objective and could represent easily recognisable markers for behavioural assessments. Finally, we analysed the repeatability of individual elephant behaviour across behavioural contexts, demonstrating the importance of incorporating a personality element in welfare assessments. Our findings highlight the potential of non-expert observers to contribute to the reliable monitoring of Asian elephant welfare across large captive working populations, which may help to both improve elephant wellbeing and safeguard human workers.

## 1. Introduction

Identifying stress in human-maintained animal populations is crucial to the development of effective welfare practices. Stress is generally defined as any threat to homeostasis or wellbeing [1,2,3], and its early detection and treatment can help to prevent numerous costs associated with chronic stress, such as the development of psychological illnesses [4], the contraction of chronic diseases [5,6] and parasites [7,8] and ultimately, increased mortality [9,10,11]. While there is reasonable information on stress in domesticated species [12,13,14], relatively little is known about the behavioural stress responses of many non-domesticated species held in captivity. Non-domesticated species are often much less familiar with humans than domesticated ones, so are likely more sensitive to human-induced stress. Amongst such non-domesticated species, Asian elephants (*Elephas maximus*) are somewhat unique. With a global population of ~45,000 individuals, up to a third exist in captivity [15,16], distributed mostly across the logging industry, the tourist trade and zoos. They exhibit cognitive capabilities and social life-histories remarkably analogous to humans and other great apes [17,18], but over 15,000 are held in captivity and managed by humans on a daily basis, a context likely to increase stress, reduce wellbeing and create a serious risk to both elephant and human safety. Alleviating human-induced stress should therefore be a central component of captive Asian elephant management, but doing so requires a welfare assessment built upon a reliable framework [19].

Elephant workers must be able to quickly, efficiently and non-invasively recognise the stress responses of elephants, so that appropriate action can be taken to safeguard both themselves and their elephants. Although there are several well-established physiological measures of stress in wildlife and zoo biology, one of the most popular being glucocorticoids [20], monitoring these can be expensive, slow and potentially inaccurate, as there will often be considerable delay between initial stressors and the subsequent collection and laboratory analysis of physiological data. With sufficient behavioural knowledge, stress can instead be assessed in real-time through the observation of stress-displacement [21] and stereotypic behaviour [22]—context-irrelevant behaviour thought to aid in coping with and regulating stress, such as pacing and repeated head bobbing in caged mammals [23]. Because of their simplicity, speed and non-invasive nature, behavioural observations are already commonly implemented in zoos and the farming industry with the use of behavioural ethograms [24,25,26], which are catalogues of species-specific behaviours. Ethograms used in zoos for assessments of Asian elephant welfare range from basic descriptions of individual behavioural events (such as ‘walk’, ‘feed’ or ‘sway’ [27]), which demand little specialist knowledge, to qualitative behavioural assessments that necessitate the integration of complex body-wide behaviour (such as ‘content’, ‘depressed’ or ‘relaxed’ [28]). The latter approach is made even more challenging because of the differences in individual personality, which can often confound the relationship between behaviour and stress [29,30].

If a behaviour is to be used as a marker of stress, it must be easily identifiable and clearly defined to maintain a high level of objectivity. However, observations carried out by multiple observers or those without sufficient behavioural knowledge are likely to be less accurate than those from a single experienced observer. For behavioural assessments of stress to be applied globally and across captive contexts, by Asian elephant workers who possess varying degrees of behavioural knowledge, its reliability (the consistency of assessment) for multiple non-specialist observers should be tested [31,32]. While this has been carried out for relatively small populations of zoo elephants [33], it has yet to be explored in captive working populations, the largest of which is used in the timber extraction process [16]. Compared to zoo elephants who are confined within an artificial space, logging elephants may exhibit a wider repertoire of natural behaviours as they work, feed and socialise in and around their natural habitat. However, they may also experience work-related stress associated with the type of human control and interaction that is absent in the conditions found in zoos. The average age and experience of elephant workers employed in the logging industry has decreased over the last 20 years [34], so the potential for less knowledgeable observers to contribute to elephant welfare assessment is pertinent to guiding the future management of these populations.

Using behavioural observations collected by different non-specialist observers, we evaluated the universal application of behavioural observations for welfare assessment in a large population of semi-captive Asian elephants working in the timber industry in Myanmar. To do this, video footage of over 100 elephants completing a behavioural task was collected, where they were tasked with picking up a known and novel object in separate phases to elicit different responses. From this footage, we have built a new ethogram of work- and potential welfare-related behaviours, designed to incorporate all characteristic behaviour exhibited during the task. This ethogram was then used by three observers with no prior knowledge of elephant behaviour research or management, to record behaviour from 217 task videos. We analysed the reliability of these observations (i) between-observers, to measure the magnitude of overall agreement by all observers, and (ii) within-observers, to measure how consistent individuals were at identifying behaviour. Further, to explore the importance of individual personality in the assessment of elephant welfare, we (iii) tested the repeatability of elephant behaviour between known and novel object tests. Our results assess the degree to which non-specialist observers can effectively recognise elephant behaviour, and therefore, whether they can be reliably utilised to monitor elephant welfare across captive populations.

## 2. Materials and Methods

### 2.1. Study Population

The Republic of the Union of Myanmar is home to the largest captive population of Asian elephants in the world. More than half of these are owned by the Myanma Timber Enterprise (MTE), who utilise the draught power of over 2700 elephants for timber extraction. Each MTE elephant is assigned a dedicated mahout after taming, who rides, directs and cares for their elephant, potentially over the elephant’s entire lifetime [34]. This individual care is supplemented by a team of veterinarians, who document regular health checks and general life history information in each elephant’s personal logbook [35,36]. Despite being maintained by humans, MTE elephants are considered semi-captive rather than fully captive, because of certain aspects of their management. Outside of working hours (year-round) and for most of the day and night during the hot season (March–May), elephants are released to roam unsupervised in the surrounding habitat, where they forage in their social groups and may encounter and mate with wild conspecifics. Pregnant females are given maternity leave for around two years, commencing half-way through pregnancy until their calf reaches one year of age, with their calf remaining at their side in relative freedom for around four years before beginning the taming process. Additionally, any elephant who reaches the age of 55 is retired but their mahouts continue to care for them until death. Therefore, although MTE elephants are likely exposed to heightened stress levels exacerbated by a human-controlled working environment [37,38], these management practices are responsible for allowing MTE elephants to closely resemble wild elephants in terms of mortality rate [39], reproductive life history [40] and social behaviour [18,41].

### 2.2. Collection of Behavioural Observations

In March and April of 2017 and 2018, we filmed 104 MTE elephants (54 female and 50 male) using a Sony HDR-CX405 video camera completing an object pick up task. This was an experiment modified from previous studies on rodents and primates (see [42] for a review), in which a known and novel stimulus is used to create contrasting environments that elicit differing behavioural responses. Here, elephants were presented with a control and novel object in separate phases, the phase order being randomised between elephants, and were commanded by their individual mahout, who was riding them, to pick the objects up. This situation is analogous to the daily working environment of MTE elephants, in which they would be required to handle and manipulate timber under the command of their mahout. However, the use of tools (such as hooks) by the mahouts was not allowed during the task, meaning the elephants were not punished for refusing commands. Although a potentially restrictive context under which to evaluate behaviour, this task standardises the collection of behavioural data and allows comparisons to be easily made between animals, dates and locations. We used two novel objects: a water bottle in 2017, which represented a novel appearance, feel and sound when manipulated, and a green plastic disc in 2018, which represented a novel shape and feel. We also used small pieces of nearby timber as control objects as they were familiar to the elephants. In total, we recorded 235 single object pick up videos (118 known and 117 novel) across 118 individual task events, including repeated observations for 14 individuals that were tested in both years. All tasks were carried out during the morning, when it was cooler, to minimise temperature-induced stress, in timber camps located in Katha and Kawlin, in the Sagaing Region of Myanmar. Behavioural research practices were approved by national governmental authorities and the ethical board of the University of Turku.

To quantify the behaviour of elephants in response to the object pick up task, we compiled distinctive behaviours from the videos into an ethogram of elephant behaviour (Table 1). To measure the magnitude of overall agreement between observers when making observations using this ethogram (inter-observer reliability), we employed three volunteer biology students from the University of Sheffield (observers A, B and C). At the time of data collection, students were in their first or second year of undergraduate study, and though they will have been taught about general theories of animal behaviour and behavioural ecology, they had no prior experience in elephant behaviour research or specialist training in stress behaviour. These observers coded the frequency of ethogram behaviours observed in the videos, using the Behavioural Observation Research Interactive Software (BORIS version 7 [43]). To familiarise observers with the behaviours and video coding software, they first went through an initial training phase. This began with an introductory meeting involving a discussion of the ethogram (with example video clips) and a practise run-through of the coding software, after which the observers were asked to code 18 videos in their own time (questions via email were encouraged). This data was compared to observations made by the lead author, so that general feedback on their recognition of ethogram behaviours could be provided; for example, at what point to count smaller ear movements as an *ear flap* event. A second round of practise video coding was originally planned following this feedback, but observer performance was deemed sufficient to move directly onto the next stage. After training, observers then independently coded the remaining 217 videos of 100 elephants (a total of 651 observations; initial observations were omitted from all analyses). Observers were asked to not discuss their observations with each other.

After video coding, a random subset of 21 videos (19 videos for observer A because of a file handling error; ~10% of all videos included in the main analyses) were generated for each observer and were then re-coded to measure how consistent individuals were at identifying behaviour (within-observer reliability). All behavioural observations began within one second of object presentation and terminated either one second after the object was released from the elephant if picked up, or after the elephant showed little to no interest in the object (mean video time = 20 s, range = 4–57 s).

### 2.3. Statistical Analysis

All statistical analyses were carried out in R version 3.6.0 [44], using the frequency of ethogram behaviours coded in each video for count-type behaviours, and duration for *testing time* and *holding time*.

#### 2.3.1. Inter-Observer Reliability

To test the reliability of behavioural observations between volunteers, we assessed the inter-observer reliability for each behaviour with a two-way random, consistency, average-measures intraclass correlation coefficient (*ICC*; [45]) using the *irr* package in R [46]. Inter-observer reliability allows the degree of agreement between independent observers to be quantified, and through this analysis, results can be generalised to other observers who possess a similar degree of elephant behaviour knowledge or observational experience [47]. We used previously established thresholds to interpret *ICC*s [48], with observer agreement deemed poor for *ICC* < 0.4, moderate for 0.4 ≤ *ICC* < 0.6, good for 0.6 ≤ *ICC* < 0.75 and excellent for *ICC* ≥ 0.75.

#### 2.3.2. Within-Observer Reliability

To test whether individual volunteers were consistent in their identification of behaviour, we analysed the reliability of repeated observations made on a subset of 19–21 videos by each observer with a two-way random, consistency, single-measures *ICC*, using the *irr* package in R and previously stated *ICC* agreement thresholds. This was calculated separately for each behaviour per observer, but mean *ICC*s were also calculated within-observers to produce mean-observer *ICC* scores, so that results could be easily compared between observers.

#### 2.3.3. Elephant Behaviour Repeatability

We then assessed the repeatability of elephant behaviour between known and novel object types, from 109 known and 108 novel object tasks (with 10 elephants tested in both years; a further four elephants were omitted as their videos were used in the initial training phase). Models were fitted using the *rptR* package in R [49], which allowed us to calculate repeatability while also incorporating a generalised linear-mixed model structure. Adjusted *R*^2^ values were generated for each behaviour at the level of the random effect of elephant identity, after accounting for the fixed effects of object type and observer identity, and uncertainty in fitted models was evaluated using 1000 bootstraps (link-approximated *R*^2^ will be presented for generality). Distributions we used in repeatability models for each behaviour can be found in Table S1 with *testing time* and *holding time* (Table 1b) being rounded to the nearest integer so that a Poisson model could be fitted. *Trunk in mouth* and *object test with foot* (Table 1) behaviours were modelled using a binary distribution, as they were only observed occurring more than once by a single observer in a single video. Following *ICC* thresholds, the repeatability of behaviour was considered poor for *R*^2^ < 0.4, moderate for 0.4 ≤ *R*^2^ < 0.6, good for 0.6 ≤ *R*^2^ < 0.75 and excellent for *R*^2^ ≥ 0.75.

## 3. Results

The ethogram constructed from object pick up tasks performed by 104 elephants included six general elephant behaviours found in zoo welfare assessment material [28] and the elephant gestures database ([50]; Table 1a), and eight object-directed behaviours relevant to the working life of MTE elephants (Table 1b). Because of the restricted (yet standardised) nature of the object pick up task, only a limited number of behaviours were incorporated into this ethogram, with behaviour involved in other contexts (such as feeding and sociality) being excluded. Of the general behaviours, trunk curl-drop is highly characteristic and most closely resembles a mixture of ‘trunk-twisting’ and ‘trunk-curl’ from the gestures database, however, descriptions sufficiently matching this behaviour could not be found.

Across the 651 total video observations carried out by the three observers of 217 pickup task videos, the most commonly observed of all 14 tested ethogram behaviours was the object-directed behaviour *object pick up*, which occurred in 81.6% of observations (Table S2). Conversely, the least common object-directed behaviour was *object test with foot*, which was only observed in 6.3% of all observations. Of the general behaviours, the most commonly present was *ear flap*, which occurred at least once in 258 out of 651 observations (39.5%), and the least common was *trunk in mouth*, which was only present in 18 out of 651 videos on average (2.9%) and was also the least common of all ethogram behaviours. The frequency of behaviours appeared to differ between object types (Table S3), with *testing time* being longer and elephants being twice as likely to exhibit *object in mouth* and *object test with foot* behaviours when presented with the novel object.

### 3.1. Inter-Observer Reliability

The reliability of observations made between observers was excellent for 9 out of 14 ethogram behaviours (64%), with four behaviours being good (29%) and only one being moderate (7%; Table 2; see Table S2 for behaviour frequencies). No behaviours fell in the poor range (*ICC* < 0.4), suggesting the level of inter-observer agreement was reasonably high across behaviours, and that the majority of behaviours defined in our ethogram should therefore be sufficiently recognisable by non-specialist observers. *Object pick up*, a simple binary behavioural event, was highly recognisable with an *ICC* of 0.939 (indicating that ~94% of observational data can be explained by similarities between observers; all three observers agreed on its presence or absence in 201 out of 217 videos). In contrast, *object test with foot* had the lowest reliability amongst ethogram behaviours (*ICC* = 0.567), with ~43% of variation due to observer differences. Although there was agreement between all three observers on the frequency of *object test with foot* in 191 out of 217 videos, this included 188 videos where this behaviour was not observed at all, meaning only in three videos did all observers agree it was present.

### 3.2. Within-Observer Reliability

On average, the reliability of repeated observations made by individual observers ranged from *ICC* = 0.836–0.964 (Table 3; see Table S4 for behaviour frequencies), with 83% of behaviours falling in the excellent range across observers. This implies that observers largely recorded the same behavioural events when videos were observed twice, and therefore observers were consistent in their identification and definition of the majority of ethogram behaviours. The only behaviour detected with poor within-observer reliability was *object test with trunk* for observer B with an *ICC* of 0.320 and therefore a repeated observation error of 68% in this instance. There was large variation in observer performance however, with observer C being the most consistent and recording the same behavioural frequency across repeated observations (*ICC* = 1.000) for six ethogram behaviours. A number of behaviours were not observed in the smaller subset of 19–21 videos used in this analysis, and there were enlarged *ICC* confidence intervals for certain behaviours recorded by observers A and B.

### 3.3. Elephant Behaviour Repeatability

When accounting for object type and observer identity, the repeatability of elephant behaviour varied between known and novel object tasks depending on which behaviour was assessed. For six behaviours, repeatability across object types was excellent, for one it was good, for five it was moderate and for the remaining two it was poor (Table 4; see Table S2 for behaviour frequencies). Behaviours exhibiting high repeatability were potentially more closely associated with within-individual variation in behaviour than those with lower repeatability. For example, *object pick up* was highly repeatable (*R*^2^ = 0.953), suggesting the probability that an elephant picked up an object was highly associated with consistent individual behavioural patterns, irrelevant of the type of object stimulus and after accounting for differences in observational data between observers. Conversely, *testing time* was only somewhat repeatable (*R*^2^ = 0.584), meaning a large proportion of the variation in the time an elephant took to pick up the object cannot be explained by elephant personality, and may be better explained by another factor.

## 4. Discussion

Behavioural observations are commonly utilised in the welfare assessment of captive Asian zoo elephants [33], but here we evaluated their reliability in the context of larger human-employed working populations, using a sizable dataset of 100 elephants. To assess the reliability of behavioural observations for elephant workers possessing various levels of behavioural knowledge, we tested observations carried out by non-specialist observers. From behavioural observations made on 217 videos of 100 elephants, our study has shown that with relatively rapid training, three observers with no prior experience in elephant research or management can reliably identify a series of behaviours relevant to the daily working-life and welfare of MTE elephants. By evaluating the objectivity of behavioural observations using two approaches of estimating reliability, we have shown that observers not only independently agreed on the frequency of all ethogram behaviours at a moderate-to-excellent level (*ICC* > 0.4), but through repeated observation, they also appeared to be consistent in their recognition of behavioural events. Further, our findings can be generalised to other observers who possess a similarly low level of specialist knowledge [47], showing that newly employed elephant workers have the potential to effectively identify Asian elephant behaviour and contribute to the monitoring of their welfare with little training. Though it could be argued that our observers, being educated biology students, possessed considerably more knowledge of animal behaviour than someone with no scientific training, compared to working mahouts this knowledge is largely theoretical and lacking in any practical or theoretical specialist experience with elephants and/or stress behaviour. Nevertheless, it is possible that our observers still outperform workers entering the mahout and zoo keeper professions without the same formal training or education, so the generality of our results must be considered when extrapolating to other contexts. Further, to ensure observers can reliably identify behaviour across different conditions, such as social interactions, behaviour from non-working contexts should also be tested.

The differences between the frequency of behaviour exhibited between object types (Table S3), while not tested statistically here, supports the notion that the object pick up task did indeed elicit differential behavioural responses. The valency of behavioural responses (their intrinsic ‘goodness’ or ‘badness’) remains unknown at this point, but elephants may react to the novel object with either neophobia, becoming increasingly stressed when commanded to approach and pick up the object, or neophilia, becoming interested in the object and eager to explore/play with it. Being a long-lived, highly intelligent and social species, Asian elephants may exhibit both types of behaviour towards novel stimuli [51]: initially behaving with neophobia to avoid the unknown risks associated with unidentified objects, followed by increasingly explorative neophilia. In our study, elephants took longer to test the novel object on average and held onto it for a shorter time than the known object (Table S3), suggesting they are acting with caution and may perceive the object as a threat. Conversely, elephants also exhibited the behaviours *object in mouth* and *stand on/kick* at a higher rate during novel object tasks on average, behaviour that is indicative of object-exploration or play behaviour. However, the frequency of *object testing with trunk* and *object testing with foot* behaviours, two exploratory behaviours related to object uncertainty, was low (Table S2), suggesting elephants may not have perceived the object stimuli as unusual—although low inter-observer reliability for these behaviours (Table 2) may imply that this low rate was associated with behaviour identification difficulties. It is unclear whether Asian elephants tend towards neophobia or neophilia, and their response will likely be influenced by their individual personality [52]. To therefore test the valency of behavioural responses, and to identify key behavioural indicators of elephant welfare, the relationship between this ethogram and physiological measures of stress should be examined. It would also be an interesting area for future studies to further explore elephant behaviour when presented with novel objects.

### 4.1. Inter-Observer Reliability

To examine the degree to which different observers agreed on the presence of behaviour, we first analysed the reliability of observations made between observers, a common approach used for testing behavioural and personality assessments [28,53,54]. There was excellent agreement between observers for the majority of behaviours, suggesting our ethogram (Table 1) was overall highly objective and the behaviours included in it represent easily recognisable indicators for behavioural assessments. Although *object test with foot* had the lowest inter-observer reliability, this behaviour may be one of the most easily misidentified from the ethogram—it is described in a way that intends to incorporate the directional, back-and-forth movements characteristic of object uncertainty, but this means it can resemble many other movements, such as when elephants make multiple pick up attempts or when their mahout is trying to position them near the object. Similarly, *trunk curl*, *trunk curl-drop* and *object test with trunk* may resemble multiple other trunk movements to non-specialist observers, such as reaching above the head to interact with a riding mahout, moving to avoid contact with passing elephant workers, or sniffing and exploring the terrain. The final behaviour with lower inter-observer reliability, *trunk in mouth*, is theoretically a very distinguishable behaviour, but in reality may have become mixed up with *object in mouth*.

To mitigate confusion around these more nuanced behaviours, further training and a discussion of behavioural definitions with observers could be undertaken. However, as behaviour becomes more complex and interwoven with environmental variables, such as social interactions, the integration of contextual information may be essential. This would require expert knowledge of Asian elephant behavioural ecology, so could only be performed by an observer with sufficient practical experience and would likely be less quantitative than behavioural coding. Qualitative behavioural assessments are already routinely carried out for several species, most frequently those involved in the farming industry [13,55,56] as their welfare is particularly threatened [57], but their reliability has recently been tested in African and Asian zoo elephants [28]. Of the final six behavioural components considered by Yon et al. [28], three were deemed highly reliable between observers (‘playful’, ‘wary’ and ‘at ease in the environment’), but three linked strongly to welfare were deemed sufficiently unreliable (‘distressed’, ‘fearful’ and ‘attentive’). Whilst qualitative behavioural assessments are more likely to be unreliable (inconsistent between observers [58]), as they require more abstract observations that are open to opinion, they have the potential to evaluate welfare quickly and efficiently [59]. Nevertheless, this approach ultimately necessitates the participation of an experienced behavioural specialist [59,60] who, while not uncommon in the zoo community, are less readily available when spread across the much larger working populations of Southeast Asia. Although MTE mahouts are very skilled and knowledgeable in the everyday handling of their elephants, they are often lacking in formal scientific training and younger, less experienced mahouts might not be able to properly interpret behaviour. This is even more important as employment age and work experience in elephant workers employed in the logging industry over the last 20 years have been decreasing [34] For this reason, qualitative measures of behaviour were not included in this study, as we aimed to explore the utility of non-specialist observations so that results could be generalised to elephant workers of any experience level.

### 4.2. Within-Observer Reliability

To further test the objectivity of behavioural observations, we also chose to explore the reliability of repeated observations made within observers, a less common but equally important method of reliability testing [31,32,58]. High inter-observer reliability has the potential to mask variation in individual observer performance, and therefore when used on its own, studies could be missing vital information on the level of understanding by participating observers. In this study, average within-observer reliability was high for all observers, but there were noticeable differences amongst observers, with observer C identifying behaviour much more consistently than observers A and B. This highlights the general existence of variation in observer ratings present in almost all multi-rater observational studies [60], which could be minimised by providing tailored individual training following the monitoring of observer performance. In our study, the reliability of repeated observations made by observers A and B for several behaviours (such as *trunk swing*) cannot be effectively assessed using the available data, as they have abnormally large confidence intervals that span three or more *ICC* thresholds. This is likely a result of the smaller number of videos utilised in this analysis (19–21 videos observed twice), which significantly limited the probability that these fairly rare behaviours would be observed (e.g., *trunk swing* was only observed in 8.9% of the total 217 videos; Table S2). When mean-observer *ICC* is restricted to behaviours that had confidence intervals spanning two or fewer thresholds, within-observer reliability for observers A and B increased to a similar level as observer C, with *ICC*s of 0.952 and 0.948, respectively.

### 4.3. Elephant Behaviour Repeatability

To assess the importance of a personality component in behaviour, we analysed the repeatability of individual elephant behaviour between known and novel object tests, while controlling for object type and observer identity. Six behaviours were highly repeatable, suggesting they could be related to individual consistency in behaviour [61], and conversely, two had poor repeatability, suggesting they were not explained by individual consistency and may instead be better explained by object type or another unknown factor. As with within-observer reliability, repeatability could not be properly assessed for four behaviours as confidence intervals spanned three or more thresholds, likely because of the low number of task comparisons made per elephant (each elephant took part in an average of 1.1 known and 1.1 novel tasks). *Object pick up* and *holding time* were highly repeatable, showing that elephants who picked objects up did this regularly for both types of objects, and the length of time elephants held on to objects was consistent across tasks. This may be the result of a high level of individual consistency in object pickup behaviour, but it could also be explained by individual-variation in elephant training. The responsiveness of elephants to their mahouts is linked to their receptiveness to training, which is further confounded by their underlying behavioural and social tendencies. Responsiveness in itself may therefore actually represent an aspect of personality; accordingly, in this population a personality component describing the responsiveness of elephants towards mahouts, ‘attentiveness’, has already been described [54]. Several behaviours such as *trunk swing* and *ear flap* were also highly related to individual consistency, so the presence of these may not act as informative behavioural assessment indicators unless individual personality is also considered [29]. Further, the propensity to experience stress can also be interpreted as a personality component, with individuals reacting differently to potentially stressful stimuli, so identifying these highly susceptible individuals in captive populations is important to long-term management considerations. Behaviours such as *trunk curl* and *trunk in mouth* were much less repeatable and may therefore act as more simplistic welfare indicators if elephant personality is not known. Personality is a measure of consistent behavioural patterns within an individual across time and context [13], and although elephants were observed across two behavioural contexts (the known and novel object task) in our study, only data from 10 elephants across both test years were analysed. Therefore, ours represents a preliminary test of the relationship between behaviour and personality—more observations from elephants tested across multiple years are needed before any empirical conclusions can be drawn.

## 5. Conclusions

Evaluating the reliability of behavioural observations is a vital first step in constructing a behaviour-based welfare assessment, and here we have shown that observers may require very little previous experience with the animal species to collect meaningful behavioural data. All three observers in our study were in high agreement for the frequency of most behaviours included and were consistent in their identification of behaviour under repeated video exposure. Now it has been shown to be reliable, our ethogram (Table 1) must next be validated with physiological stress data to establish evidence-based links between behaviour and welfare. This framework needs to incorporate individual personality, as our analysis indicates that behaviours may be more, or less, related to differences in individual expression across contexts, depending on the behaviour in question.

For the largest captive population of Asian elephants in the world, the welfare of almost 3000 elephants must be consistently monitored across a large forested region. This is a demanding task, but our results demonstrate that observers with no significant knowledge of elephant behaviour can reliably catalogue behavioural indicators of elephant welfare, meaning welfare assessors from a wide pool of educational backgrounds could therefore be effectively utilised. Further, though MTE mahouts are becoming younger and less experienced over time [34], they are still likely to be able to reliably contribute to the monitoring of their elephants’ welfare, helping to promote safer, more ethical and effective human–elephant working relationships.

## Figures and Tables

**Table 1:** Ethogram of Myanma Timber Enterprise (MTE) elephant behaviour during the object pick up task.

(**a**) General behaviour	
**Behaviour**	**Description**
Trunk swing	Directional swinging of trunk with a back and forth motion, including side to side or forward and back
Trunk curl	Curling or coiling of the trunk (without immediate collapsing)
Trunk curl-drop	Trunk is partially raised, curled around itself and collapsed down
Trunk in mouth	Trunk is put into or held in the elephant’s mouth
Ear flap	Both ears are fully extended in a rapid movement (ear movements caused by riding mahouts not counted)
Tail flick	Tail is flicked in one direction with the tip exceeding 90º from resting position
(**b**) Object-directed behaviour	
**Behaviour**	**Description**
Object pick up	Object is picked up at least once with the elephant’s trunk
Object in mouth	Object is put into or held in the elephant’s mouth
Throw/flick	Throwing or flicking of the object with the elephant’s trunk (excluding when object is dropped)
Stand on/kick	Object is stood on or kicked
Object test with trunk	Trunk is extended towards the object and then removed
Object test with foot	Foot is extended towards the object and then removed
Testing time	Duration an elephant is exposed to the object without picking it up
Holding time	Duration an elephant is holding the object with its trunk or mouth

**Table 2:** Inter-observer reliability and 95% confidence intervals for ethogram behaviours. General behaviours (Table 1a) above the dotted line and object-directed behaviours (Table 1b) below.

Behaviour	*ICC*	95% Confidence Intervals
Trunk swing	0.882	0.852–0.907
Trunk curl	0.686	0.603–0.754
Trunk curl-drop	0.609	0.504-0.694
Trunk in mouth	0.661	0.575–0.732
Ear flap	0.964	0.955–0.972
Tail flick	0.897	0.850–0.927
Object pick up	0.939	0.924–0.952
Object in mouth	0.9	0.871–0.922
Throw/flick	0.92	0.896–0.938
Stand on/kick	0.959	0.949–0.968
Object test with trunk	0.7	0.474–0.813 *
Object test with foot	0.567	0.456–0.658
Testing time	0.997	0.996–0.998
Holding time	0.994	0.993–0.995

* Confidence interval spans two or more thresholds.

**Table 3:** Within-observer reliability, 95% confidence intervals and mean-*ICC* for each observer. General behaviours (Table 1a) above the dotted line and object-directed behaviours (Table 1b) below.

Behaviour	Observer A		Observer B		Observer C	
	*ICC*	95% Confidence Interval	*ICC*	95% Confidence Interval	*ICC*	95% Confidence Interval
Trunk swing	0.634	0.267–0.841 *	0.459	0.032–0.741 *	0.836	0.644–0.930
Trunk curl	0.697	0.371–0.871 *	0.837	0.642–0.930	0.935	0.814–0.975
Trunk curl-drop	0.578	0.177–0.814 *	-	-	-	-
Trunk in mouth	0.654	0.304–0.85 *	-	-	1.000	1.000–1.000
Ear flap	0.879	0.713–0.952	0.987	0.968–0.995	1.000	1.000–1.000
Tail flick	0.888	0.723–0.956	0.977	0.944–0.991	0.984	0.962–0.994
Object pick up	1.000	1.000–1.000	0.882	0.737–0.950	1.000	1.000–1.000
Object in mouth	1.000	1.000–1.000	1.000	1.000–1.000	1.000	1.000–1.000
Throw/flick	0.946	0.869–0.979	0.944	0.869–0.977	1.000	1.000–1.000
Stand on/kick	0.940	0.854–0.976	-	-	-	-
Object test with trunk	0.930	0.828–0.973	0.320	0.000–0.658 *	0.932	0.840–0.972
Object test with foot	-	-	-	-	1.000	1.000–1.000
Testing time	0.988	0.971–0.995	0.981	0.950–0.992	0.997	0.992–0.999
Holding time	0.997	0.992–0.999	0.976	0.941–0.990	0.885	0.743–0.951
Mean-*ICC*	0.856		0.836		0.964	

* Confidence interval spans two or more thresholds.

**Table 4:** Repeatability and error estimates for elephant behaviour. General behaviours (Table 1a) above the dotted line and object-directed behaviours (Table 1b) below.

Behaviour	Adjusted *R*^2^	Standard Error	95% Confidence Interval
Trunk swing	0.894	0.034	0.931–1.000
Trunk curl	0.431	0.083	0.235–0.556
Trunk curl-drop	0.483	0.233	0.000–0.754 *
Trunk in mouth	0.530	0.050	0.790–0.972
Ear flap	0.873	0.048	0.789–0.968
Tail flick	0.851	0.047	0.758–0.940
Object pick up	0.953	0.011	0.959–0.996
Object in mouth	0.567	0.113	0.325–0.751 *
Throw/flick	0.665	0.105	0.450–0.846 *
Stand on/kick	0.864	0.052	0.827–0.998
Object test with trunk	0.249	0.056	0.117–0.344
Object test with foot	0.185	0.186	0.082–0.709 *
Testing time	0.584	0.045	0.485–0.664
Holding time	0.769	0.037	0.697–0.844

* Confidence interval spans two or more thresholds.

## Supplementary Materials

The following are available online at https://www.mdpi.com/2076-2615/10/1/167/s1, Table S1: Distributions used in repeatability modelling. General behaviours (Table 1a) above the dotted line and object-directed behaviours (Table 1b) below, Table S2: The presence, average and total range per video of ethogram behaviours across the 651 total video observations carried out by the three observers used for inter-observer reliability and behaviour repeatability. The range stated is the lowest and highest number of behaviours per video recorded by any observer. General behaviours (Table 1a) above the dotted line and object-directed behaviours (Table 1b) below, Table S3: The frequency of ethogram behaviours exhibited per video between object types. Values have been averaged between observers and standardised against video length. General behaviours (Table 1a) above the dotted line and object-directed behaviours (Table 1b) below, Table S4: The presence, average and total range per video of ethogram behaviours per observer (for within-observer consistency testing). General behaviours (Table 1a) above the dotted line and object-directed behaviours (Table 1b) below.
