# Supplementary material for: Evaluating the Reliability of Non-Specialist Observers in the Behavioural Assessment of Semi-Captive Asian Elephant Welfare
Source: Animals (Basel). 2020 Jan 18;10(1):167. doi: 10.3390/ani10010167 (PMC7022305; doi:10.3390/ani10010167)
Supplement: Supplementary file 1 [file animals-10-00167-s001.pdf]

animals

Supplementary Materials: Evaluating the Reliability of Non-Specialist Observers in the Behavioural Assessment of Semi-Captive Asian Elephant Welfare

Jonathan L. Webb [1,2,*], Jennie A. H. Crawley [3], Martin W. Seltmann [3], Océane Liehrmann [3,4], Nicola Hemmings [1], U Kyaw Nyein [5], Htoo Htoo Aung [5], Win Htut [5], Virpi Lummaa [3] and Mirkka Lahdenperä [3]

1 Department of Animal and Plant Sciences, University of Sheffield, Sheffield S10 2TN, United Kingdom; jonathan.wb@outlook.com (J.L.W.); n.hemmings@sheffield.ac.uk (N.H.)
2 School of Psychology, University of Auckland, 23 Symonds Street, Auckland 1010, New Zealand; jonathan.wb@outlook.com (J.L.W.)
3 Department of Biology, University of Turku, 20500 Turku, Finland; jahcra@utu.fi (J.A.H.C.); martin.seltmann@utu.fi (M.W.S.); oceane.liehrmann@gmail.com (O.L.); virpi.lummaa@utu.fi (V.L.); mirkka.lahdenpera@utu.fi (M.L.)
4 UFR des Sciences, University of Caen, 14000 Caen, France; oceane.liehrmann@gmail.com (O.L.)
5 Myanma Timber Enterprise, Gyogone Forest Compound, Bayint Naung Road, Insein Township, Yangon 11011, Myanmar; kyaw.nyein.mte@gmail.com (U.K.N.); kohtooaung723@gmail.com (H.H.A.); winhtut641@gmail.com (W.H.)
* Correspondence: jonathan.wb@outlook.com

Received: 29 November 2019; Accepted: 10 January 2020; Published: date

Table S1. Distributions used in repeatability modelling**.** General behaviours (Table 1A) above the dotted line and object-directed behaviours (Table 1B) below.

Behaviour	Datatype
Trunk swing	Poisson
Trunk curl	Poisson
Trunk curl-drop	Poisson
Trunk in mouth	Binary
Ear flap	Poisson
Tail flick	Poisson
Object pick up	Binary
Object in mouth	Poisson
Throw/flick	Poisson
Stand on/kick	Poisson
Object test with trunk	Poisson
Object test with foot	Binary
Testing time	Poisson
Holding time	Poisson

Table S2. The presence, average and total range per video of ethogram behaviours across the 651 total video observations carried out by the three observers used for inter-observer reliability and behaviour repeatability. The range stated is the lowest and highest number of behaviours per video recorded by any observer. General behaviours (Table 1A) above the dotted line and object-directed behaviours (Table 1B) below.

Behaviour	Presence (%)	Average per video	Range
Trunk swing	8.9	0.14	0-5
Trunk curl	28.7	0.44	0-6
Trunk curl-drop	8.0	0.10	0-4
Trunk in mouth	2.9	0.03	0-2
Ear flap	39.5	1.10	0-11
Tail flick	37.6	1.39	0-22
Object pick up	81.6	-	-
Object in mouth	16.6	0.18	0-3
Throw/flick	17.4	0.24	0-5
Stand on/kick	11.5	0.16	0-4
Object test with trunk	45.3	0.66	0-6
Object test with foot	6.3	0.06	0-2
Testing time	-	11.06 s	1-57 s
Holding time	-	5.54 s	0-37 s

Table S3. The frequency of ethogram behaviours exhibited per video between object types. Values have been averaged between observers and standardised against video length. General behaviours (Table 1A) above the dotted line and object-directed behaviours (Table 1B) below.

Behaviour	Known	Novel
Trunk swing	0.008	0.004
Trunk curl	0.016	0.025
Trunk curl-drop	0.005	0.005
Trunk in mouth	0.008	0.004
Ear flap	0.060	0.053
Tail flick	0.070	0.066
Object pick up	0.990*	0.952*
Object in mouth	0.014	0.004
Throw/flick	0.017	0.015
Stand on/kick	0.004	0.011
Object test with trunk	0.025	0.042
Object test with foot	0.004	0.002
Testing time	0.422	0.639
Holding time	0.446	0.268

*Values indicate the observer-averaged probability of object pick up

Table S4. The presence, average and total range per video of ethogram behaviours per observer (for within-observer consistency testing). General behaviours (Table 1A) above the dotted line and object-directed behaviours (Table 1B) below.

Behaviour	Observer A			Observer B			Observer C		
	Presence (%)	Average per Video	Range	Presence (%)	Average per video	Range	Presence (%)	Average per Video	Range
Trunk swing	18.4	0.29	0–3	9.5	0.1	0–1	20	0.17	0–1
Trunk curl	31.6	0.61	0–4	26.2	0.36	0–2	54.8	0.88	0–6
Trunk curl-drop	23.7	0.24	0–1	0	0	0	0	0	0
Trunk in mouth	7.9	0.08	0–1	0	0	0	4.8	0.05	0–1
Ear flap	50	1.08	0–6	28.6	0.95	0–7	38.1	1.48	0–10
Tail flick	28.9	0.84	0–11	23.8	1.02	0–9	47.6	2.48	0–15
Object pick up	68.4	-	-	73.8	-	-	90.5	-	-
Object in mouth	10.5	0.16	0–2	23.8	0.24	0–1	14.3	0.14	0–1
Throw/flick	7.9	0.18	0–3	21.4	0.31	0–2	19	0.19	0–1
Stand on/kick	18.4	0.29	0–2	0	0	0	0	0	0
Object test with trunk	28.9	0.42	0–4	23.8	0.26	0–2	69	1.36	0–6
Object test with foot	0	0	0	0	0	0	9.5	0.1	0–1
Testing time	-	14.13	2–56	-	9.74	1–46	-	10.93	2–38
Holding time	-	5.66	0–21	-	5.76	0–26	-	6.26	0–13
